# Complete Genome Sequence of *Bacillus* sp. Strain IGA-FME-1, Isolated from the Bulk Soil of Maize (*Zea mays* L.)

**DOI:** 10.1128/MRA.00192-21

**Published:** 2021-04-01

**Authors:** Zhenhua Yu, Sergio de los Santos-Villalobos, Junjie Liu, Xiaobing Liu, Fannie Isela Parra Cota, Valeria Valenzuela Ruiz, Guanghua Wang

**Affiliations:** a Key Laboratory of Mollisols Agroecology, Northeast Institute of Geography and Agroecology, Chinese Academy of Sciences, Harbin, China; b Instituto Tecnológico de Sonora, Ciudad Obregón, Sonora, Mexico; c Campo Experimental Norman E. Borlaug, Instituto Nacional de Investigaciones Forestales, Agrícolas y Pecuarias, Ciudad Obregón, Sonora, Mexico; University of Rochester School of Medicine and Dentistry

## Abstract

Here, we present the complete genome of *Bacillus* sp. strain IGA-FME-1 (isolated from the bulk soil of maize [*Zea mays* L.]). This genome consists of 5,147,837 bp, 5,219 protein-coding genes, 112 tRNAs, 13 16S rRNAs, 13 23S rRNAs, and 13 5S rRNAs, with a G+C content of 38.2%.

## Abstract (precis)

Here, we present the complete genome of *Bacillus* sp. strain IGA-FME-1 (isolated from the bulk soil of maize [*Zea mays* L.]). This genome consists of 5,147,837 bp, 5,219 protein-coding genes, 112 tRNAs, thirteen 16S rRNAs, thirteen 23S rRNAs, and thirteen 5S rRNAs, with a G+C content of 38.2%.

## ANNOUNCEMENT

Maize (*Zea mays* L.) is the third most significant cereal worldwide, after rice and wheat (1). It covers one-third of China's harvesting area, and more than 200 million tons of maize are produced annually in that country (2). Cereal crops, such as maize, can associate with many species of plant growth-promoting rhizobacteria (PGPR), resulting in grain yield increases, as well as greater aerial biomass production (3). The *Bacillus* species are widely studied PGPR (4, 5), with reported salt stress amelioration (6), zinc solubilization (7), salt stress tolerance (8), drought tolerance (9), indole and siderophore production, and phosphate solubilization (10). In addition, this genus provides biological control against the pathogens Macrophomina phaseolina, Fusarium moniliforme, and Fusarium graminearum, causal agents of maize rot (11).

The *Bacillus* strain IGA-FME-1 was isolated from the bulk soil of maize in an agricultural field of Lishu, Jilin, China (43°20′N, 124°28′E), by using a serial dilution method with solid Luria-Bertani (LB) culture medium at 28°C for 2 days (12). After purification, this strain was cryopreserved at −80°C using LB culture medium and 30% glycerol. For genome sequencing, the IGA-FME-1 strain was incubated in liquid LB culture medium for 48 h at 28°C at 180 rpm, followed by centrifugation (10,000 × *g* for 10 min), and the supernatant was discarded. High-quality genomic DNA was extracted using the E.Z.N.A. bacterial DNA kit (Omega Bio-tek, USA), according to the manufacturer’s instructions.

The quality and quantity of the extracted genomic DNA were determined using 1% agarose gel electrophoresis and a Qubit 4.0 fluorometer. High-quality DNA (optical density at 260 nm [OD_260_]/OD_280_, 1.8 to 2.0; total DNA amount, ≥1 μg; concentration, ≥20 ng/μl) was used for further sequencing. For PacBio sequencing, a Covaris g-TUBE was used to shear the DNA, followed by damage repair, end repair, blunt-end adaptor ligation, and size selection. Size selection was performed by using the BluePippin system and the set size cutoff threshold. Then, AMPure PB magnetic beads were used to purify and select DNA fragments to construct a SMRTbell library (SMRTbell template kit, version 1.0). DNA sequencing was performed by using the PacBio (Menlo Park, CA, USA) RS II platform. The quality of the raw reads obtained was analyzed by FastQC version 0.11.5 (13). Trimmomatic version 0.32 (14) was used to remove adapter sequences and low-quality bases; only 1.95% of reads were dropped. Subsequently, a *de novo* assembly was generated by SPAdes version 3.14.1 (15), using the parameters --careful for error correction in reads and --cov-cutoff auto (in which SPAdes automatically computes the coverage threshold by using a conservative strategy). The IGA-FME-1 genome size is 463,187,922 bp with 50,423 subreads, with an average length of 9,186 bp; the subread *N*_50_ was 9,721 bp. Subsequently, a *de novo* assembly was generated using Flye (16), and the best genome assembly result was selected, followed by circularization of the genome using Circlator (17) to identify and trim the genome overlap to confirm that the genome was closed. The final assembly consisted of 5,147,837 bp under one scaffold, with a G+C content of 38.2%.

Genome annotation was performed by the NCBI Prokaryotic Genome Annotation Pipeline (PGAP) (18). The genome is predicted to contain 5,219 protein-coding genes, 112 tRNAs, 13 16S rRNAs, 13 23S rRNAs, 13 5S rRNAs, 1 copy of a transfer-messenger RNA, and 105 miscellaneous RNAs, with a G+C content of 38.2%. Default parameters were used for all software unless otherwise noted.

### Data availability.

This draft genome sequence has been deposited in DDBJ/ENA/GenBank under accession number CP064793.1. The version described in this paper is the first version, under BioProject number PRJNA668551 and BioSample number SAMN16414965. Raw data have been deposited in the NCBI SRA under accession number PRJNA678836. The complete genome sequence of *Bacillus* sp. strain IGA-FME-1 (BioSample number SAMN16814809) is available under accession number SRX9518585.
